# A low chromium diet increases body fat, energy intake and circulating triglycerides and insulin in male and female rats fed a moderately high-fat, high-sucrose diet from peripuberty to young adult age

**DOI:** 10.1371/journal.pone.0281019

**Published:** 2023-01-26

**Authors:** Jesse Bertinato, Philip Griffin

**Affiliations:** 1 Nutrition Research Division, Bureau of Nutritional Sciences, Health Products and Food Branch, Health Canada, Ottawa, Ontario, Canada; 2 Department of Biochemistry, Microbiology and Immunology, University of Ottawa, Ottawa, Ontario, Canada; Universidad de Extremadura Facultad de Enfermeria y Terapia Ocupacional, SPAIN

## Abstract

Trivalent chromium (Cr) may function to potentiate the action of insulin, but the effects of chromium intakes on metabolic parameters are unclear. Cr is listed as a potentially beneficial element for rodents based on studies that show feeding low quantities affect glucose metabolism. Cr is recommended at 1 mg per kg in rodent diets. This study examined the effects of different levels of dietary Cr on body weight, body composition, energy intake, food efficiency and metabolic parameters of lipid and glucose metabolism in male and female rats when fed from peripuberty to young adult age in the background of a moderately high-fat, high-sucrose diet. Sprague-Dawley CD rats (n = 10 males and 10 females/group) at 35 days of age were assigned by weight to the low (LCr, 0.33 ± 0.06 mg/kg), normal (NCr, 1.20 ± 0.11 mg/kg) or high (HCr, 9.15 ± 0.65 mg/kg) Cr diets. Diets were fed ad libitum for 12 weeks (83 days). At baseline, body weights and composition were similar (p≥0.05) among diet groups. Compared to the NCr group, the LCr group weighed more (p<0.01) and consumed more energy (food) from Day 56 onwards, but food efficiency was unaffected. Following an oral glucose challenge (Day 77), dietary chromium levels did not affect plasma glucose, but fasting plasma insulin and insulin at 30 and 60 min after dosing were higher in the LCr group compared to the NCr group. At the end of the study, whole-body fat, accrued body fat from baseline and fasting serum triglycerides were higher in the LCr group compared to the NCr group. Effects were similar in both sexes and not observed in the HCr group. These data show that low dietary Cr affects metabolic parameters common in chronic diseases underscoring the need for clinical trials to define the nutritional and/or pharmacological effects of Cr.

## Introduction

The National Academy of Medicine considers trivalent chromium (Cr) an essential nutrient with a function in potentiating the action of insulin [1]. A more recent review (2014) by the European Food Safety Authority Panel on Dietetic Products, Nutrition and Allergies concluded that the essential function of Cr in metabolism has not been substantiated and studies aimed at inducing Cr deficiency in animals have produced inconsistent results [2]. Dietary Reference Values for Cr were not established for the European Union because it was judged that there is no evidence of essentiality of Cr in animal nutrition or of beneficial effects associated with Cr intake in healthy people.

The American Institute of Nutrition (AIN) Ad Hoc committee lists Cr as a “potentially” beneficial ultra trace element for rodents [3]. More recent work conflicts earlier findings regarding a biological role for Cr, and thus, its nutritional role has not been firmly established. The AIN-93G rodent diet for growth, pregnancy and lactation and the AIN-93M diet for adult maintenance recommends addition of Cr at 1 mg per kg diet, a recommendation based on results from studies showing that feeding low levels of Cr has negative effects on glucose metabolism [3].

Earlier studies in male rats conducted in the 1990’s reported that a low Cr diet causes overproduction of insulin in response to a glucose challenge [4–6]. In those studies, the diets were formulated to impair glucose metabolism by increasing fat and sugar content and altering concentrations of essential minerals such as copper and iron. A more recent study in male rats, without dietary stressors, reported no differences in the Area Under the Curve (AUC) for plasma insulin levels in a glucose tolerance test between rats fed a very low Cr diet (16 μg/kg diet) or a diet containing the AIN-93G recommended amount for 23 weeks [7]. In that study dietary Cr did not affect body weight or food consumption, similar to results reported in other rat studies [5, 6, 8].

Animal and in vitro experiments have suggested a number of putative mechanisms by which Cr may modulate insulin action and regulate glucose metabolism including increasing the kinase activity of insulin receptor β, enhancing the activity of downstream effectors of insulin signaling phosphoinositide 3-kinase and protein kinase B, downregulation of negative-regulators of insulin signaling, upregulation of AMP-activated protein kinase, regulation of membrane cholesterol efflux and increasing glucose transporter-4 translocation to the cell surface [9]. However, at present the effects of Cr intakes on metabolic parameters such as body weight, body composition, energy (food) intake and markers of lipid and glucose metabolism are unclear because studies in rats [4–8] and in humans [10–18] have produced inconsistent results. Differences in the study group or animal model, length of the study, Cr doses, Cr source (compound) and background diet may partly explain the inconsistent results. Also, effects of Cr may be small and undetectable in studies that are not adequately powered or rigorously conducted.

Over one-quarter of the adult US population consumes a supplement containing Cr [19]. Cr is added to infant formula and in Canada can be added to supplemented foods at a maximum level of 125 μg/serving with cautionary statements [20]. Dietary Cr may affect the development and progression of chronic diseases such as obesity, diabetes and metabolic syndrome by modulating insulin action and glucose metabolism, but conclusive evidence that Cr deficiency alters metabolic parameters is lacking [21]. Thus, this study examined the effects of a low and high Cr diet on body weight, body composition, energy intake, food efficiency, lipid profile and markers of glucose metabolism in rats fed a moderately high-fat, high-sucrose diet commonly consumed in North America [22, 23]. Peripubertal rats were chosen for this study because of their high energy needs relative to body weight to sustain rapid growth which may make them a sensitive model to detect small effects on energy intake, body weight and body composition. Sex differences in diet-induced obesity and metabolic disturbances exist [24, 25] and thus effects were examined in both sexes.

## Materials and methods

### Diets and animal protocol

Sixty (30 male and 30 female) Sprague-Dawley CD rats (Charles River Canada, St. Constant, QC, Canada) at 35 days of age were assigned (matched by body weight) to 1 of 3 isocaloric high-fat, high-sucrose diets (Dyets, Inc., Bethlehem, PA, USA). Diets were fed ad libitum for 12 weeks (83 days). Rats were started on the experimental diets in 5 groups of 12 rats (2 males and 2 females per diet group) over 5 successive days to facilitate technical manipulations during the study. The low (LCr), normal (NCr) or high (HCr) Cr diets were formulated using the AIN-93G mineral mix [3] without Cr. Cr was added to the NCr and HCr diets from a Cr(III) potassium sulfate dodecahydrate premix in sucrose. The NCr diet was formulated to contain normal amounts of Cr (1 mg/kg diet) based on the AIN-93G recommendations [3]. By analysis, the diets contained 0.33 ± 0.06 mg/kg (LCr), 1.20 ± 0.11 (NCr) and 9.15 ± 0.65 (HCr) mg of Cr per kg of wet diet (S1 Table). Compared to the standard AIN-93G diet, the experimental diets were high-energy diets (4396 vs. 3766 kcal/kg) and carbohydrate, fat and protein were 51%, 33% and 16% of total energy compared with 64.0%, 16.7% and 19.3% for the standard diet, respectively. Sucrose was added at 290.1 g/kg compared to 100 g/kg in the standard diet and simple sugars were 28% of energy. Diets were a modified version of Research Diets, Inc. D12266B formulation used previously to induce obesity in rats [26–28].

Rats were singly housed in plastic, solid-bottom cages with a wire-grill insert, platform and shelter. Rats were housed without bedding throughout the study. Cages were held in vented racks. Rats were kept on a 12:12-hour light:dark cycle and had free access to food and deionized water throughout the study. Food consumption and body weights were measured weekly. Food consumption was determined by weighing the feeder and determining the weight of food missing from the feeder. Food efficiency was calculated as body weight gain (g) divided by energy intake (kcal). Body composition was measured at the start (Day 0) and end (Day 83) of the study using magnetic resonance imaging (EchoMRI-4in1™ system, EchoMRI, Houston, TX, USA). During week 6 of the study, all feces were collected for 7 consecutive days. To minimize contamination of the feces, feces were carefully collected each day from an Iso-Pad (Ketchum, Brockville, CA, USA) placed under the wire-grill insert and cages were fitted with a new Iso-Pad after each collection. Feces from the 7-day collection were pooled and kept frozen at −20°C for the collection period and then stored at −80°C until analysis.

After 12 weeks of feeding the diets, rats were fasted overnight (~12 hour) and killed the following morning by exsanguination under general isoflurane anesthesia. Blood was collected from the abdominal aorta by syringe and dispensed into a Trace Element Serum tube (BD 368380, Thermo Fisher Scientific, Ottawa, Ontario, Canada). Blood was allowed to clot for 30 min at room temperature and then centrifuged (2000 × g, 10 min, 4°C) to isolate serum for analysis of lipid profile. The right leg was excised and immediately frozen on dry ice and then stored at –80˚C until analysis.

One male rat from the NCr group was euthanized during Week 8 of the study after biting and ingesting the plastic tubing during a practice oral glucose tolerance test (OGTT). One male rat from the HCr group died unexpectedly (without prior symptoms) inside the EchoMRI chamber while measuring body composition on Day 83 of the study. The experimental protocol was approved by the Health Products and Food Branch Animal Care Committee of Health Canada (Protocol No.: 2020–004).

### Oral glucose tolerance test

On Day 77 of the study an OGTT was performed on all the rats. Following an overnight fast (~12 hour), rats were weighed to calculate the dextrose dose for each rat (2 g dextrose/kg body weight). A 0.4 g/mL dextrose solution (in deionized water) was administered slowly using a syringe and plastic catheter making sure the rats were swallowing the solution. Blood samples (~250 μl) were drawn from the tail vein before dextrose dosing (0 min) and 30, 60 and 120 min after dosing. To permit rapid blood collection and minimized the stress response, rats were warmed under an infrared heat lamp for 4 min prior to blood collections and briefly restrained during the procedure with a AIMS™ Disposable Plastic Restraint. Blood samples were dispensed into BD Microtainer™ tubes with lithium heparin (BD 365965, VWR, Mississauga, ON, Canada). Tubes were immediately placed on ice and centrifuged (2000 × g, 15 min, 4°C) within 45 min to isolate plasma. Plasma samples were immediately frozen at −80˚C until measurement of glucose and insulin concentrations.

### Femur isolation and physical measurements

Femurs were isolated from the right leg. Skin and flesh were removed using a scalpel and forceps. Wet and dry femur weights were measured using a Mettler Toledo AT261 delta range analytical balance (Mettler Toledo, Mississauga, ON, Canada). Femur length from the greater trochanter to the lateral condyle and mid-diaphysis mediolateral width were measured using a digital caliper (62379–531, VWR). Femur volume was determined using a PYREX™ specific gravity bottle (01–716, Thermo Fisher Scientific). Femur volume was calculated as the mass of water displaced by the femur in grams divided by the density of water (0.99997 g/cm^3^). Femur density was calculated as the wet weight in grams divided by the volume in cubic centimeters.

### Mineral analyses

Ground diets (~1.5 g samples) and femurs were placed in preweighted quartz beakers and dried overnight at 100°C in an Isotemp® oven (Thermo Fisher Scientific). Samples were cooled, placed in a desiccator for 1 hour and then weighed to obtain dry weights. Diets and femurs were ashed using a combination of dry ashing with a Thermo Scientific Lindberg/Blue MTM box furnace (Thermo Fisher Scientific) and wet ashing using concentrated trace metal grade nitric acid (Thermo Fisher Scientific). Ashes were solubilized in ~12 mL (weighed accurately) of 1 N (diets) or 3 N (femurs) nitric acid.

Frozen feces (one week collection) were ground using disposable grinder cups with the Tube Mill 100 Control, IKA (VWR) and dried as described above. Approximately 1 g of dry feces sample (weighed accurately) was microwave digested (CEM Model: MARS 6) in 10 mL of concentrated nitric acid. Hydrogen peroxide (2 mL) was added to the samples and the samples were microwave digested a second time. The entire sample was transferred into a quartz beaker and the acid evaporated on a hotplate. The sample was dry-ashed in a box furnace and the ashes were solubilized in ~10 mL of 3 N nitric acid.

Cr in diet samples was measured using inductively-coupled plasma mass spectrometry with the NexION 300S (PerkinElmer, Inc., Woodbridge, ON, Canada) equipped with an apex-ST PFA MicroFlow nebulizer and a SC-Fast series autosampler. The instrument was controlled using the Syngistix version 1.1 software and was operated using an RF power of 1600 W and argon flow rates of 1.05, 1.2 and 18 L/min for the nebulizer, auxiliary and plasma, respectively. Analytical accuracy was verified (within 3-sigma limit) using the TM-25.3 and TMDA-61.2 Certified Reference Materials (Environment Canada, Burlington, ON, Canada). Percent recoveries for control standards were 93.7−98.9% (CVs, <3.4%).

Mineral concentrations in solubilized femur and feces ashes were measured using a radial view 700 Series inductively-coupled plasma optical emission spectrometer (Agilent Technologies Canada Inc., Mississauga, ON, Canada). Operating conditions have been described previously [29]. Standard calibration curves were prepared using the IV-Stock-4 multi-element standard (Inorganic Ventures, Christiansburg, VA, USA) for chromium, copper, iron and zinc or a PlasmaCAL custom standard (SCP Science, Baie-d’Urfé, Québec, Canada) for potassium, calcium, magnesium, phosphorus and sodium. Analytical accuracy was verified using the TMDA-61.2 and ION-96.4 Certified Reference Materials (Environment Canada). For the different minerals the CVs ranged from 0.7−6.1%.

### Assays

Plasma insulin was measured using the Rat Ultrasensitive Insulin ELISA (80-INSRTU-E01, Alpco Diagnostics, Salem, NH, USA). Level 2 and 3 controls were within the acceptable ranges (CVs, <5.8%). Plasma glucose was measured using the Glucose Colorimetric Assay Kit (10009582, Cayman Chemical, Ann Arbor, MI, USA). The CVs of control samples were <5.9%. Serum lipids were measured using the ABX Pentra 400 chemistry analyser (HORIBA Instruments Inc., Irvine, CA, USA) with the ABX Pentra Cholesterol CP, ABX Pentra HDL Direct 100 CP, ABX Pentra LDL Direct CP and the ABX Pentra Triglycerides CP reagents. Quality controls (N MultiControl and P MultiControl, Raeyco Lab Equipment Systems Management Ltd., Burnaby, BC, Canada) were within the acceptable ranges (CVs, 3.9−5.9%).

### Statistical analyses and calculations

The number of rats per diet group were informed by results from previous rat studies that investigated the effects of dietary Cr on glucose metabolism (4−8). Results are reported as means ± SD. Mixed-design ANOVA was used to determine overall effects and interactions of time, sex and diet. Univariate results are shown for each time point for parameters with a significant (p<0.05) time × sex or time × diet interaction. Two-way ANOVA was used for analysis of parameters measured at a single time point to examine overall effects and interactions of sex and diet. For parameters with a significant diet effect, Dunnett’s test was used to compare the LCr and HCr groups to the NCr group (Control). One-way ANOVA followed by Tukey’s post hoc test was used to determine differences in Cr concentrations among diets. Homogeneity of variances was assessed using Levene’s test. Data that showed unequal variances were log or Box-Cox transformed prior to analysis. AUC for glucose and insulin were calculated using the trapezoidal rule with the AUC function in SigmaPlot 12.5 (Systat Software Inc., Chicago, IL, USA). Statistical significance was set at p<0.05. Data were analyzed using Statistica 13.1 (TIBCO Software Inc., Palo Alto, CA, USA).

## Results

### Cr in diets and feces

Differences in Cr intakes among diet groups were corroborated by measuring Cr concentrations in the feces (S2 Table). The ratios of Cr concentrations in the diets were 0.27:1 (LCr:NCr) and 1:7.6 (NCr:HCr). Corresponding ratios of Cr in feces were 0.28:1 and 1:10.4 for the males and 0.26:1 and 1:9.2 for the females.

### Body weights and composition

**Fig 1:**
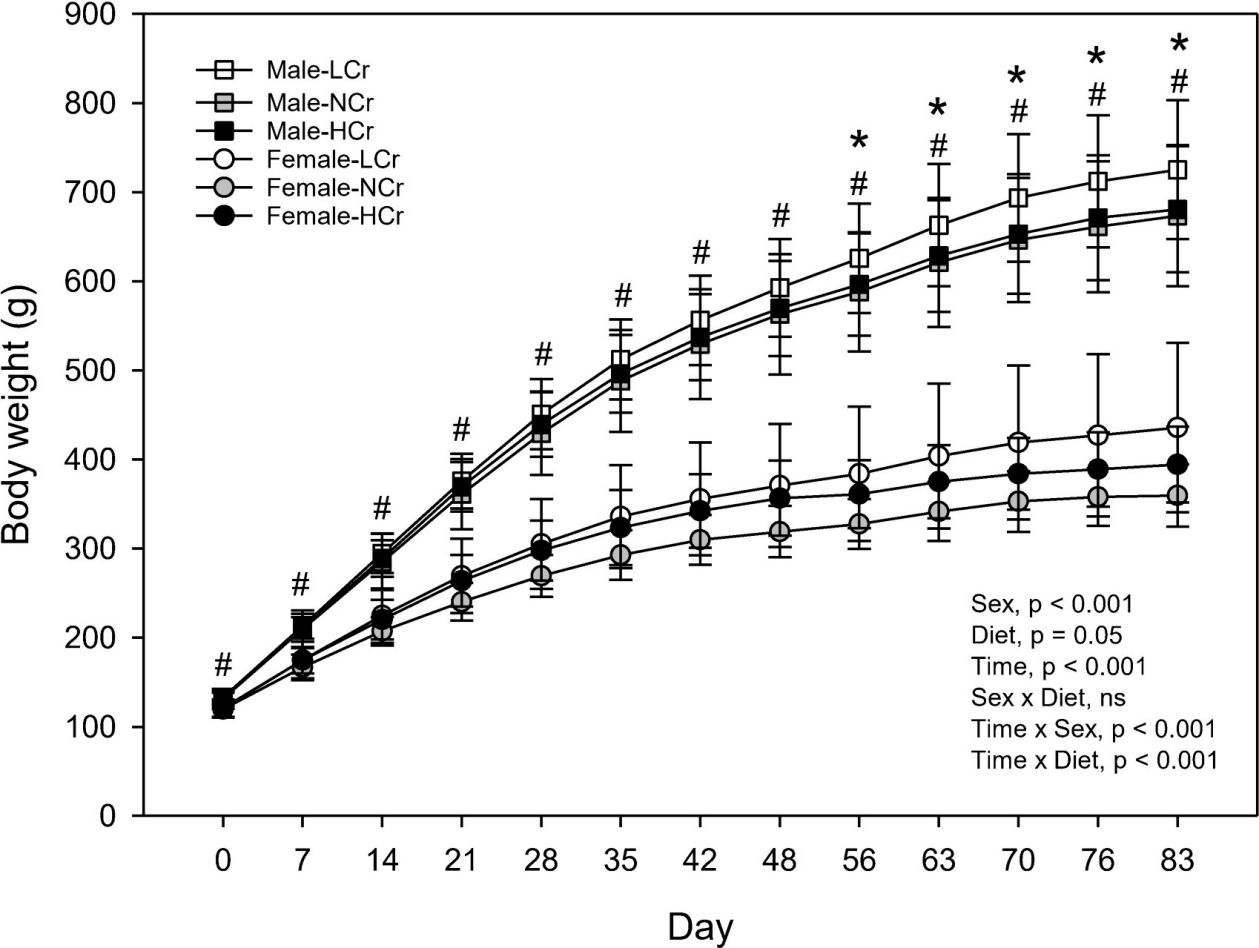
Body weights of male and female rats. Results are presented as means ± SD, n = 9−10. Results were analyzed by mixed-design ANOVA to determine effects and interactions of time, sex and diet. Time × sex and time × diet interactions (p<0.001) were observed. Univariate results are shown for each time point for effect of sex (^#^, p<0.001). For effect of diet, results of males and females were pooled and the LCr group differed from the NCr group by Dunnett’s test (*, p<0.01). ns, p≥0.05.

For all parameters, a sex × diet interaction was not detected (p≥0.05) and therefore results for male and female rats were pooled to assess the effect of diet. Male rats weighed more than female rats at all time points (Fig 1). From Day 56 to Day 83, rats fed the LCr diet weighed more than rats fed the NCr diet. At baseline (Day 0), lean mass, percent lean mass, body fat mass and percent fat mass were similar in rats fed the different diets (Table 1). On Day 83, body fat mass, accrued fat mass from baseline and percent fat mass were higher, while percent lean mass was lower in rats fed the LCr diet compared to rats fed the NCr diet.

**Table 1:** Body composition of rats.

Parameter	Males			Females			ANOVA					
	LCr (n = 10)	NCr (n = 9)	HCr (n = 10)	LCr (n = 10)	NCr (n = 10)	HCr (n = 10)	Sex	Diet	Time	Sex × Diet	Time × Sex	Time × Diet
Lean (g)^1^							p < 0.001	ns	p < 0.001	ns	p < 0.001	ns
Day 0	114 ± 5	112 ± 9	111 ± 5	103 ± 7	103 ± 6	104 ± 8	p < 0.001					
Day 83	530 ± 34	507 ± 36	510 ± 39	279 ± 27	262 ± 27	274 ± 23	p < 0.001					
Δ Day 83^2^	416 ± 31	395 ± 31	399 ± 36	176 ± 24	159 ± 23	170 ± 22	p < 0.001	ns		ns		
Lean (%)^1^							p < 0.01	0.06	p < 0.001	ns	p < 0.001	p < 0.05
Day 0	83.2 ± 1.2	82.9 ± 1.1	82.3 ± 1.1	82.7 ± 1.5	83.4 ± 0.9	82.6 ± 0.9	ns	ns		ns		
Day 83	73.5 ± 5.9*	75.6 ± 4.7	75.2 ± 5.2	65.5 ± 8.5*	73.0 ± 3.5	69.8 ± 4.6	p < 0.001	p < 0.05		ns		
Δ Day 83^2^	-9.7 ± 5.1*	-7.3 ± 4.5	-7.1 ± 4.8	-17.2 ± 7.3*	-10.5 ± 3.3	-12.8 ± 4.6	p < 0.001	p < 0.05		ns		
Fat (g)^1^							p < 0.01	p < 0.05	p < 0.001	ns	p < 0.01	p < 0.05
Day 0	17.7 ± 2.4	17.1 ± 2.5	17.9 ± 2.2	16.3 ± 2.8	15.0 ± 1.7	16.3 ± 1.7	p < 0.01	ns		ns		
Day 83	173 ± 63*	145 ± 48	149 ± 50	143 ± 74*	84 ± 16	107 ± 28	p < 0.01	p < 0.05		ns		
Δ Day 83^2^	155 ± 61*	128 ± 46	131 ± 49	127 ± 71*	69 ± 15	90 ± 28	p < 0.01	p < 0.05		ns		
Fat (%)^1^							p < 0.01	p < 0.05	p < 0.001	ns	p < 0.001	p < 0.05
Day 0	12.9 ± 1.3	12.6 ± 0.9	13.2 ± 1.0	13.0 ± 1.2	12.1 ± 0.8	12.9 ± 0.9	ns	ns		ns		
Day 83	23.4 ± 5.7*	21.1 ± 4.5	21.5 ± 5.2	31.3 ± 8.6*	23.4 ± 3.6	26.8 ± 4.6	p < 0.001	p < 0.05		ns		
Δ Day 83^2^	10.4 ± 5.0*	8.5 ± 4.1	8.4 ± 4.7	18.3 ± 7.8*	11.3 ± 3.1	13.9 ± 4.7	p < 0.001	p < 0.05		ns		

Values are means ± SD.

^1^ Analyzed by mixed-design ANOVA to determine the effects and interactions of time, sex and diet.

^2^ Analyzed by two-way ANOVA. Univariate results are shown for each time point for parameters with significant (p<0.05) time × sex or time × diet interaction. For effect of diet, results of males and females were pooled and differ from the NCr group by Dunnett’s test

(*, p<0.05). ns, p≥0.1.

### Energy intake and food efficiency

**Fig 2:**
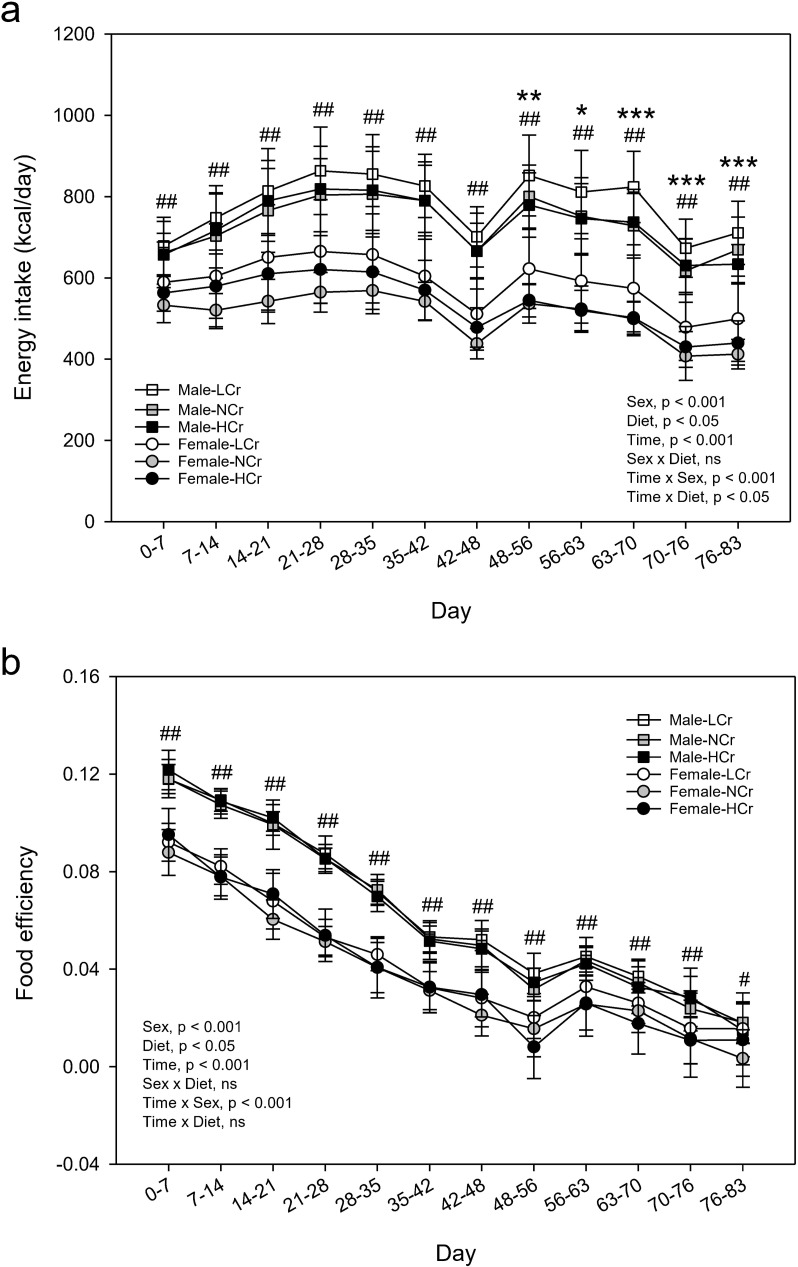
Energy intake and food efficiency of male and female rats. Results are presented as means ± SD, n = 9−10. Results for energy intake (a) and food efficiency (b) were analyzed by mixed-design ANOVA to determine effects and interactions of time, sex and diet. For energy intake, time × sex (p<0.001) and time × diet interactions (p<0.05) were observed. Univariate results are shown for each time point for effect of sex (^##^ p<0.001). For effect of diet, results of males and females were pooled and the LCr group differed from the NCr group by Dunnett’s test (*, p<0.05; **, p<0.01; ***, p<0.001). For food efficiency, a time × sex (p<0.001) interaction was observed and univariate results are shown for the effect of sex at each time point (^#^, p<0.05; ^##^ p<0.001). For effect of diet, results of males and females and all time points were pooled, p≥0.05 (ANOVA). During Days 42−48 and Days 70−76 the practice and actual oral glucose tolerance tests were performed on the rats, respectively. Food efficiency = [body weight gain (g)/energy intake (kcal)]. ns, p≥0.05.

Energy intake was higher in male rats compared to female rats at all time points (Fig 2A). Compared to rats fed the NCr diet, energy intake in rats fed the LCr diet was higher from Day 48−56 to the end of the study (Fig 2A) and cumulative food intake was higher from Day 63 (S1 Fig). Food efficiency was higher for male rats compared to female rats at all time points (Fig 2B). For food efficiency, sex × diet or time × diet interactions were not evident. After pooling results for both sexes and all time points, a diet effect was not detected (p≥0.05).

### Femur measurements

Physical measurements of femurs differed between male and female rats (Table 2). Mid-diaphysis mediolateral width was greater in rats fed the LCr diet compared to rats fed the NCr diet. Femur length:width ratio was numerically lower in rats fed the LCr diet (p = 0.08). Mineral composition of femurs differed between sexes and diet groups (S2 Table). Male rats had lower femur concentrations of copper, iron, zinc, and magnesium and higher concentrations of potassium and sodium compared to female rats. Rats fed the HCr diet had lower femur iron concentrations and higher femur sodium compared to rats fed the NCr diet. Rats fed the LCr diet had numerically lower femur zinc concentrations compared to rats fed the NCr diet (p = 0.05).

**Table 2:** Gross morphometry of rat femurs.

Parameter	Males			Females			ANOVA^1^		
	LCr (n = 10)	NCr (n = 9)	HCr (n = 9)	LCr (n = 10)	NCr (n = 10)	HCr (n = 10)	Sex	Diet	Sex × Diet
Wet weight (g)	1.56 ± 0.08	1.52 ± 0.11	1.53 ± 0.11	1.03 ± 0.08	0.98 ± 0.09	1.01 ± 0.10	p < 0.001	ns	ns
Dry weight (g)	1.00 ± 0.05	0.96 ± 0.07	0.98 ± 0.06	0.67 ± 0.04	0.64 ± 0.06	0.66 ± 0.06	p < 0.001	ns	ns
Length (mm)^2^	42.6 ± 1.1	42.8 ± 0.6	42.9 ± 0.8	37.1 ± 0.8	36.6 ± 0.9	37.0 ± 1.1	p < 0.001	ns	ns
Width (mm)^3^	4.97 ± 0.26*	4.77 ± 0.26	4.80 ± 0.17	4.10 ± 0.16*	3.98 ± 0.19	3.94 ± 0.22	p < 0.001	p < 0.05	ns
Length:width ratio	8.59 ± 0.49^§^	9.01 ± 0.47	8.94 ± 0.26	9.07 ± 0.38^§^	9.20 ± 0.33	9.42 ± 0.49	p < 0.001	p < 0.05	ns
Volume (cm^3^)	1.08 ± 0.05	1.04 ± 0.08	1.04 ± 0.08	0.68 ± 0.06	0.64 ± 0.07	0.67 ± 0.07	p < 0.001	ns	ns
Density (g/cm^3^)	1.45 ± 0.02	1.46 ± 0.01	1.47 ± 0.02	1.52 ± 0.05	1.53 ± 0.03	1.52 ± 0.03	p < 0.001	ns	ns

Values are means ± SD.

^1^ Analyzed by two-way ANOVA. For parameters with a significant (p<0.05) diet effect, results of males and females were pooled and compared with the NCr group by Dunnett’s test

(*, p<0.05;

^§^, p = 0.08).

^2^ Length from the greater trochanter to the lateral condyle.

^3^ Mid-diaphysis mediolateral width. ns, p≥0.1.

### Lipid profile

Serum lipid profile differed between sexes (Table 3). Male rats had lower serum HDL cholesterol and higher LDL cholesterol and triglycerides. Rats fed the LCr diet had higher triglycerides compared to rats fed the NCr diet.

**Table 3:** Serum lipid profile of rats.

Parameter	Males			Females			ANOVA^1^		
	LCr (n = 10)	NCr (n = 9)	HCr (n = 9)	LCr (n = 10)	NCr (n = 10)	HCr (n = 10)	Sex	Diet	Sex × Diet
Cholesterol (mmol/L)	2.29 ± 0.38	2.18 ± 0.37	2.01 ± 0.46	2.60 ± 0.56	2.11 ± 0.56	2.40 ± 0.44	0.096	ns	ns
HDL cholesterol (mmol/L)	0.55 ± 0.04	0.54 ± 0.06	0.50 ± 0.05	0.67 ± 0.08	0.57 ± 0.11	0.65 ± 0.08	p < 0.001	0.08	0.05
LDL cholesterol (mmol/L)	0.22 ± 0.04	0.20 ± 0.03	0.19 ± 0.05	0.16 ± 0.05	0.14 ± 0.04	0.16 ± 0.05	p < 0.001	ns	ns
Triglycerides (mmol/L)	1.41 ± 0.68*	1.09 ± 0.39	1.19 ± 0.59	1.39 ± 1.15*	0.60 ± 0.19	0.66 ± 0.14	p < 0.001	p < 0.05	ns

Values are means ± SD.

^1^ Analyzed by two-way ANOVA. For parameters with a significant (p < 0.05) diet effect, results of males and females were pooled and compared with the NCr group by Dunnett’s test

(*, p<0.05). ns, p≥0.1.

### OGTT

**Fig 3:**
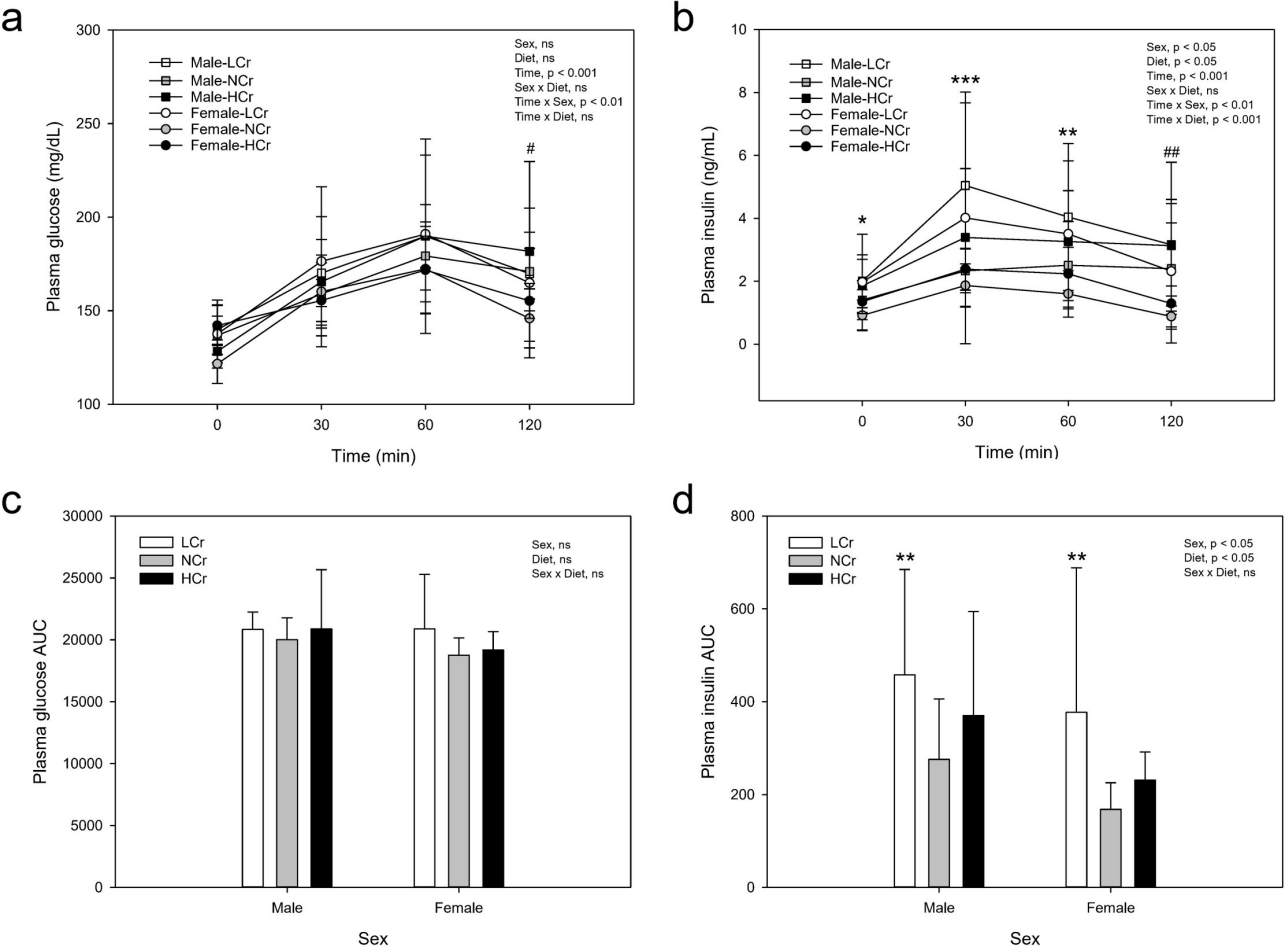
Plasma glucose and insulin responses to an oral glucose challenge conducted at Day 77 of the study. Plasma glucose (a) and insulin (b) concentrations and the respective AUC (c, d). Dextrose solution (0.4 g/mL) was orally administered to the rats (2 g dextrose/kg body weight) by gavage after an overnight fast (12 hour). Blood was collected from the tail vein before dosing (0 min) and 30, 60 and 120 min after dosing. Results are displayed as means ± SD, n = 9−10. Results were analyzed by mixed-design ANOVA to determine effects and interactions of time, sex and diet (a, b). For glucose and insulin a significant time × sex interaction (p<0.01) was observed and univariate results are shown for the effect of sex at each time point (^#^, p<0.05; ^##^, p<0.01). For insulin there was a time × diet interaction (p<0.001). For the effect of diet, results of males and females were pooled and the LCr group differed from the NCr group by Dunnett’s test (*, p<0.05; **, p<0.01; ***, p<0.001). AUC results were analyzed by two-way ANOVA (c, d). For the effect of diet, results of males and females were pooled and the LCr group differed from the NCr group by Dunnett’s test (**, p<0.01). ns, p≥0.05. AUC: Area Under the Curve.

At the 120 min time point, plasma glucose concentration was higher in male rats compared to female rats irrespective of diet (Fig 3A). AUC for glucose did not differ between sexes or among rats fed the different diets (Fig 3B). At the 120 min time point, plasma insulin concentration was higher in male rats compared to female rats irrespective of diet (Fig 3C). Rats fed the LCr diet had higher plasma insulin at baseline (0 min) and at the 30 and 60 min time points compared to rats fed the NCr diet. AUC for insulin was higher in male rats compared to female rats and in rats fed the LCr diet compared to rats fed the NCr diet (Fig 3D).

## Discussion

This study shows that a low Cr diet with approximately one-third of recommended amounts in the background of a high-fat, high-sucrose diet affects metabolic parameters common in the development and progression of chronic diseases, whereas consuming Cr above normal amounts showed no effects aside from changes in femur mineral concentrations. Rats fed the LCr diet had higher fasting triglycerides and insulin and greater AUC for insulin following an oral glucose challenge. In addition, the LCr diet increased body weights, whole-body fat mass and energy (food) consumption, which contrasts previous observations in rats [5, 6, 8]. This study also advances previous research that used only male rats and showed that the effects of the LCr diet were similar in both sexes.

Macronutrient composition of the diet may modify the effect of Cr [4, 5, 7]. Thus, the diets were formulated to have a macronutrient distribution similar to diets commonly consumed in North America [22, 23]. Results from the Canadian Community Health Survey (2015) indicated that Canadians consume 49.3%, 33.8% and 16.4% of there energy from carbohydrates, total fat and protein, respectively [23]. A high-energy diet, high in fat and sugar has been shown to induce hyperphagia [30], adiposity [27, 28] and impair glucose metabolism [31–33] in rats which may magnify the effects of Cr. A diet high in simple sugars has been shown to increase urinary Cr excretion and may increase Cr requirements [34].

The HCr diet only affected bone (femur) mineral concentrations; causing higher sodium concentrations and lower iron concentrations compared to the NCr diet. Cr may compete with iron for binding to transferrin, a main iron transport protein [35]. Very high Cr diets have been shown to alter essential mineral nutrients in tissues of female rats [36]. In that study, diets with 500−1000 mg Cr/kg decreased serum, liver and kidney iron concentrations. Bone marrow is the site of erythrocyte synthesis and a major iron storage site [37]. This study showed that a much lower Cr diet (~9 mg/kg) decreases iron concentration in bone.

Most previous studies in rats examined effects of dietary Cr only in males [4–8]. Sex and species differences exist in the development of diet-induced obesity and metabolic complications [24, 25]. Male rats have greater diet-induced hyperhagia and more metabolic complications [25]. In this study male rats weighed more, had more body fat, accrued more body fat during the study and had higher energy intakes and food efficiency, whereas female rats had higher percentage body fat and a greater change in percent body fat from baseline. In male rats serum HDL cholesterol was lower and LDL cholesterol, triglycerides and AUC for insulin following a glucose challenge were higher. Despite these sex differences, for all parameters examined, a diet × sex interaction was not detected indicating that the effects of consuming the low Cr diet were similar in both sexes.

Rats fed the LCr diet consumed more energy and, as expected, weighed more and accumulated more body fat. Food efficiency was not affected; rats fed the LCr and NCr diets gained a similar amount of body weight per kcal of food energy consumed throughout the study. Differences in energy consumption were not statistically different until Day 48−56, approximately at the same time point body weights began to differ. Differences in energy intakes between rats fed the LCr or NCr diets were likely too small to detect at earlier time points given the large variability in energy consumption between rats. As the study advanced, the higher energy intakes led to progressively larger body weights for rats fed the LCr diet which further increased the difference in energy intakes to a degree that was statistically significant by Day 48−56. Together, these results suggest that the effect of the LCr diet on energy consumption was small which may explain why effects on food consumption and body weights were not reported in previous studies with young rats fed a low Cr diet for a similar or longer period [4, 5, 7]. Energy intakes, body weights and composition were similar in rats fed the HCr or NCr diets indicating that a diet with a larger, pharmacological dose of Cr did not affect these parameters. These results are in agreement with another study that reported no effects on body mass or composition in rats administered large gavage doses of Cr [8].

Femur length was not affected by the LCr diet which is further evidence that linear growth and lean body mass was unaffected. Percent lean mass was lower because the rats weighed more. Mid-diaphysis femur width was larger in rats fed the LCr diet. This is likely explained by greater mechanical load caused by the larger body weights of the rats. Femoral structural geometry is known to adapt to mechanical loading [38]. A similar observation was made in rats fed diets with different amounts of magnesium that induced differences in body weights [28].

The risk of type 2 diabetes was shown to be lower in US adults taking Cr-containing supplements [19] and a systematic review of randomized controlled trials suggested significantly improved glycemia in people with type 2 diabetes with Cr supplementation [12]. However, a study that used improved meta-analytical methods concluded no effect of Cr supplementation on fasting glucose in people with or without type 2 diabetes [11]. Previous studies in rats reported that lower Cr intakes result in greater insulin secretion in response to an intravenous glucose challenge [5, 6]. The results were interpreted as Cr having a role in regulating glucose metabolism. With lower Cr intakes, insulin sensitivity may be diminished resulting in a compensatory overproduction of insulin to regulate blood glucose. Rats fed the LCr diet had higher baseline fasting plasma insulin and higher insulin at 30 and 60 min following an oral glucose challenge. AUC for insulin was also greater. Fasting plasma glucose at baseline and at all time points did not differ suggesting that the higher insulin secretion adequately compensated for the lower insulin sensitivity. Notably, research suggests that insulin may be an important regulator of hunger and food consumption through affects on the brain [39–43].

Compared to rats fed a standard Cr diet, Di Bona et al. [7] reported lower AUC for insulin following a glucose challenge for rats fed a Cr-supplemented diet (i.e., an additional 1 mg Cr/kg diet), but not a low Cr diet. The low Cr diet in that study did not affect AUC for insulin despite the level of Cr in the diet being ~20-fold lower compared to the LCr diet used in this study (16 μg/kg vs. 330 μg/kg). The substantially higher background Cr measured in the LCr diet in this study is likely explained by higher endogenous Cr concentrations in diet ingredients and possibly Cr contamination from (stainless steel) equipment during mixing and pelleting of the diets [44].

Metabolic syndrome is a cluster of factors of cardiovascular disease and type 2 diabetes that include abdominal obesity, hypertension, impaired fasting glucose, high triglycerides and low HDL cholesterol. In a 23-year follow-up study, the incidence of metabolic syndrome among young US adults was inversely and longitudinally associated with toenail Cr and the association was largely explained by its relation to blood lipids [45]. Rats fed the LCr diet showed a poorer lipid profile with higher serum triglycerides. Serum cholesterol, LDL cholesterol or HDL cholesterol were not different.

Even though Cr was proposed as an essential trace element in mammals more than six decades ago [21, 46] its function as an essential nutrient remains contentious [2]. Adequate Intakes for Cr range from 11 μg/day for young children 1−3 years of age to 45 μg/day for lactating women [1]. Because humans may require little Cr from the diet, overt symptoms of Cr deficiency are rarely observed; overt symptoms have only been purported in patients on total parenteral nutrition with no Cr [47–49]. Recommended amounts of Cr for rats is much higher (>100-fold) than for humans on a per body weight basis, though humans may absorb Cr more efficiently than rats [50]. Given the large difference in Cr requirements, rat studies provide limited insight into levels of Cr intakes that may induce a nutritional deficiency or pharmacological effects in humans. Nonetheless, rat studies can provide insight on the physiological processes and metabolic parameters affected by Cr intakes and modifying effects of dietary components.

This was a rigorously conducted study in both sexes with accurate measurement of food consumption and body composition using magnetic resonance imaging. Since dietary Cr is poorly absorbed (<2−5%) [1, 50] with most being excreted in the feces, concentrations of Cr in the feces was used to corroborate that Cr intakes reflected the Cr content in the diets. Cr(III) potassium sulfate dodecahydrate was used as the Cr source which eliminates ambiguity that a moiety of the Cr source (e.g., picolinate, malate) influenced the results by increasing Cr absorption or some other mechanism [51–53].

This study has some limitations. Currently, there is a poor understanding of Cr requirements for rats and our “normal” Cr diet was based on AIN-93G recommendations. The effects were observed in rats fed diets high in fat and sucrose from young age to maturity which may have increased Cr requirements and exaggerated the effects. Given that obesity and dysregulation of lipid and glucose metabolism are manifestations in chronic diseases such as diabetes and metabolic syndrome [54, 55], these results underscore the need for clinical trials to define levels of Cr intake that affect metabolic parameters of health in humans. Trivalent Cr appears to be relatively non-toxic with a large margin of safety [56] and thus substantially larger doses than current Adequate Intakes [1] can be safely tested. Overall diet (background Cr content and macronutrient composition) and developmental life-stage should be considered when designing these studies and interpreting their results. Research on delineating the mechanisms of action of Cr will also be important [9]. Hexavalent Cr is a toxic environmental contaminant [57]. With our analytical method we could not distinguish between trivalent and hexavalent Cr. Sensitive biomarkers of Cr status are lacking [58] and thus physiological Cr status of the rats was not reported. Cr concentrations in femur did not differ among diet groups indicating that femur Cr was not a sensitive enough measure to detect the differences in Cr intakes over 12 weeks.

## Conclusions

This study adds to the body of evidence on the effects of dietary Cr intakes on metabolic parameters of health. A low Cr diet (based on rodent standards) negatively affected metabolic parameters of lipid and glucose metabolism and increased energy intake, body weight and whole-body fat mass in male and female rats. These results underscore the need to examine these parameters in clinical trials that will help assess the nutritional and/or pharmacological effects of Cr.

## Supporting information

S1 Table Diet compositions. (PDF)

S2 Table Mineral content in rat femurs and feces. (PDF)

S1 Fig Cumulative food consumption of male and female rats. (PDF)

S1 Dataset (XLSX)
