# Supplementary material for: A low chromium diet increases body fat, energy intake and circulating triglycerides and insulin in male and female rats fed a moderately high-fat, high-sucrose diet from peripuberty to young adult age
Source: PLoS One. 2023 Jan 26;18(1):e0281019. doi: 10.1371/journal.pone.0281019 (PMC9879406; doi:10.1371/journal.pone.0281019)
Supplement: S1 Table — (PDF) [file pone.0281019.s001.pdf]

S1 Table. Diet compositions.

Component	Diets		
	LCr	NCr	HCr
Fixed ingredients (g/kg)[1]	709.9	709.9	709.9
Sucrose (g/kg)	290.1	289.1	280.1
Energy density (kcal/kg)[2]	4396 (18.4)	4396 (18.4)	4396 (18.4)
CHO (% kcal)[3]	51 (61)	51 (61)	51 (61)
Lipid (% kcal)[3]	33 (16)	33 (16)	33 (16)
Protein (% kcal)[3]	16 (19)	16 (19)	16 (19)
Cr premix (g/kg)[4]	–	1	10
Cr content (mg/kg DW)[5]	0.35 ± 0.06^{c}	1.29 ± 0.12^{b}	9.79 ± 0.69^{a}
Cr content (mg/kg WW)[5]	0.33 ± 0.06^{c}	1.20 ± 0.11^{b}	9.15 ± 0.65^{a}

[1] Fixed ingredients (g/kg diet): cornstarch, 202.191; casein, 190; corn oil, 118; dyetrose, 75; anhydrous milkfat, 44.2; AIN-93G mineral mix without Cr (No.: 216272), 35; cellulose (microcrystalline), 30; AIN-93G vitamin mix (No.: 310025), 10; L-cystine, 3; choline bitartrate, 2.5; ethoxyquin, 0.009. [2] Energy density in MJ/kg is shown in parentheses. [3] Percent by weight is shown in parentheses. [4] Cr premix (1 mg/g sucrose). [5] Analyzed content. Values are means ± SD, n=4. Values not sharing a common superscript letter differ by one-way ANOVA followed by Tukey's post hoc test, $p < 0.05$. CHO: carbohydrate; Cr: chromium; DW: dry weight; WW: wet weight.
