# Supplementary material for: A low chromium diet increases body fat, energy intake and circulating triglycerides and insulin in male and female rats fed a moderately high-fat, high-sucrose diet from peripuberty to young adult age
Source: PLoS One. 2023 Jan 26;18(1):e0281019. doi: 10.1371/journal.pone.0281019 (PMC9879406; doi:10.1371/journal.pone.0281019)
Supplement: S2 Table — (PDF) [file pone.0281019.s002.pdf]

S2 Table. Mineral content in rat femurs and feces.

Parameter	Males			Females			ANOVA[1]		
	LCr (n=10)	NCr (n=9)	HCr (n=9)	LCr (n=10)	NCr (n=10)	HCr (n=10)	Sex	Diet	Sex × Diet
Femur									
Cr (µg/g DW)	3.83 ± 0.15	3.77 ± 0.19	3.90 ± 0.12	3.65 ± 0.29	3.76 ± 0.33	3.83 ± 0.23	ns	ns	ns
Cu (µg/g DW)	3.59 ± 1.08	3.30 ± 0.43	3.36 ± 0.63	4.03 ± 0.36	4.09 ± 0.42	3.98 ± 0.26	p < 0.001	ns	ns
Fe (µg/g DW)	49.1 ± 9.8	59.4 ± 13.0	48.1 ± 12.1**	67.5 ± 19.4	73.3 ± 18.5	56.5 ± 7.0**	p < 0.001	p < 0.05	ns
Zn (µg/g DW)	224 ± 13§	242 ± 17	242 ± 12	264 ± 21§	268 ± 14	281 ± 14	p < 0.001	p < 0.01	ns
K (mg/g DW)	2.30 ± 0.16	2.31 ± 0.17	2.34 ± 0.14	2.10 ± 0.29	2.07 ± 0.23	1.92 ± 0.18	p < 0.001	ns	ns
Ca (mg/g DW)	284 ± 7	287 ± 6	290 ± 7	288 ± 5	285 ± 5	288 ± 5	ns	ns	ns
Mg (mg/g DW)	3.99 ± 0.23	3.98 ± 0.16	4.04 ± 0.18	4.29 ± 0.18	4.41 ± 0.20	4.36 ± 0.18	p < 0.001	ns	ns
P (mg/g DW)	137 ± 3	137 ± 3	138 ± 4	138 ± 2	135 ± 3	136 ± 2	ns	ns	ns
Na (mg/g DW)	7.58 ± 0.23	7.59 ± 0.24	7.82 ± 0.19*	7.28 ± 0.18	7.18 ± 0.19	7.32 ± 0.17*	p < 0.001	p < 0.05	ns
Feces									
Cr (µg/g DW)	4.1 ± 0.8***	14.8 ± 1.7	154.7 ± 16.8***	4.4 ± 0.7***	16.9 ± 2.3	155.9 ± 14.6***	0.06	p < 0.001	ns

Values are means ± SD. [1] Analyzed by two-way ANOVA. For parameters with a significant ($p < 0.05$) diet effect, results of males and females were pooled and compared with the NCr group by Dunnett's test (*, $p < 0.05$; **, $p < 0.01$; ***, $p < 0.001$; §, $p = 0.05$). ns, $p \geq 0.1$. DW: dry weight.
