# Supplementary material for: A low chromium diet increases body fat, energy intake and circulating triglycerides and insulin in male and female rats fed a moderately high-fat, high-sucrose diet from peripuberty to young adult age
Source: PLoS One. 2023 Jan 26;18(1):e0281019. doi: 10.1371/journal.pone.0281019 (PMC9879406; doi:10.1371/journal.pone.0281019)
Supplement: S1 Fig — (PDF) [file pone.0281019.s003.pdf]

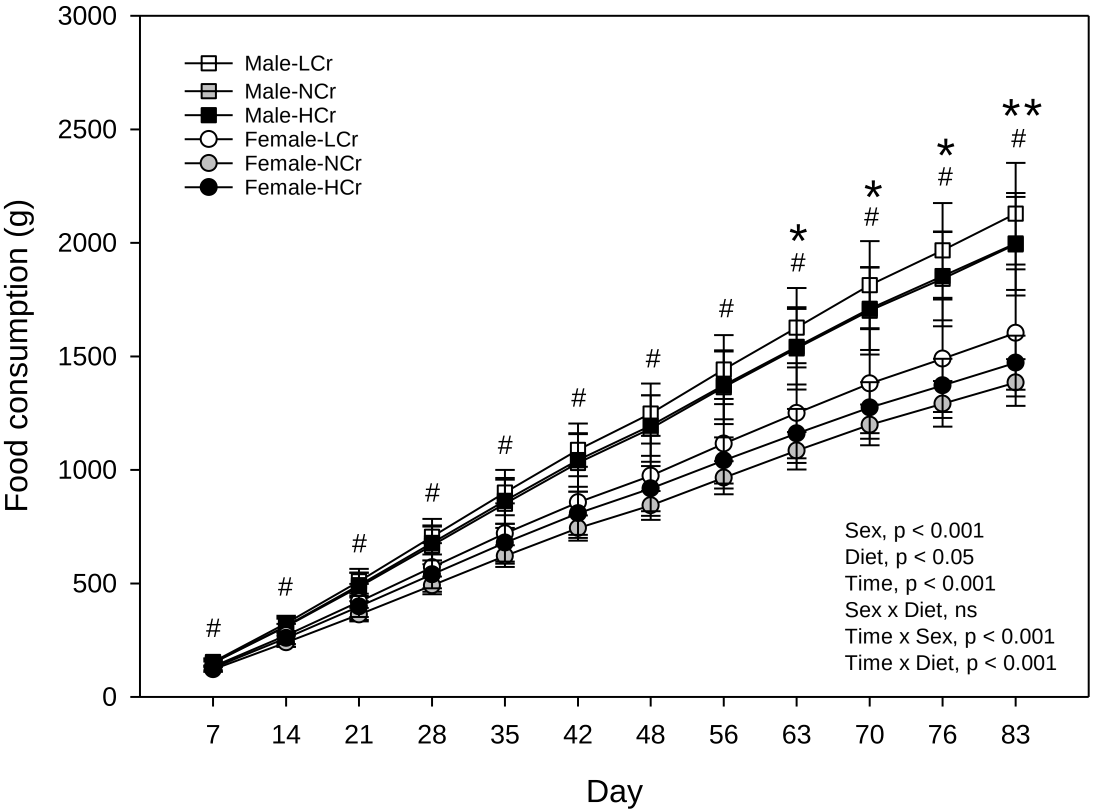

S1 Fig. Cumulative food consumption of male and female rats. Results are presented as means ± SD, n = 9–10. Results were analyzed by mixed-design ANOVA to determine effects and interactions of time, sex and diet. Time × sex and time × diet interactions ($p < 0.001$) were observed. Univariate results are shown for each time point for effect of sex ($^{\#}$, $p < 0.001$). For effect of diet, results of males and females were pooled and the LCr group differed from the NCr group by Dunnett's test (*, $p < 0.05$; **, $p < 0.01$). ns, $p \geq 0.05$.
